# The Use of Adipose-Derived Progenitor Cells and Platelet-Rich Plasma Combination for the Treatment of Supraspinatus Tendinopathy in 55 Dogs: A Retrospective Study

**DOI:** 10.3389/fvets.2016.00061

**Published:** 2016-09-09

**Authors:** Sherman O. Canapp, Debra A. Canapp, Victor Ibrahim, Brittany Jean Carr, Catherine Cox, Jennifer G. Barrett

**Affiliations:** ^1^Veterinary Orthopedic and Sports Medicine, Annapolis Junction, MD, USA; ^2^Regenerative Orthopedic and Sports Medicine, Washington, DC, USA; ^3^Marion duPont Scott Equine Medical Center, Virginia-Maryland College of Veterinary Medicine, Virginia Tech, Leesburg, VA, USA

**Keywords:** supraspinatus tendinopathy, mesenchymal stem cells, platelet-rich plasma, adipose-derived stem cells, regenerative medicine, rotator cuff

## Abstract

**Objective:**

To report clinical findings and outcomes for 55 dogs with supraspinatus tendinopathy (ST) treated with adipose-derived progenitor cells and platelet-rich plasma (ADPC-PRP) therapy.

**Methods:**

Medical records of client-owned dogs diagnosed with ST that were treated with ADPC-PRP combination therapy were reviewed from 2006 to 2013. Data collected included signalment, medical history, limb involvement, prior treatments, physical and orthopedic examination, objective temporospatial gait analysis findings, diagnostic imaging results (radiography, magnetic resonance imaging, musculoskeletal ultrasonography), arthroscopy findings, and outcome.

**Results:**

Following ultrasound-guided injection of ADPC-PRP, objective gait analysis was available on 25 of the 55 dogs at 90 days post ADPC-PRP therapy. Following treatment, a significant increase in total pressure index percentage (TPI%) was noted in the injured (treated) forelimb at 90 days post treatment (*p* = 0.036). At 90 days following treatment, 88% of cases had no significant difference in TPI% of the injured limb to the contralateral limb. The remaining 12% of cases had significantly improved (*p* = 0.036). Bilateral shoulder diagnostic musculoskeletal ultrasound revealed a significant reduction in tendon size (CSA) in the treated tendon at 90 days following treatment when compared to the initial CSA (*p* = 0.005). All cases showed significant improvement in fiber pattern of the affected supraspinatus tendon by the ultrasound shoulder pathology rating scale.

**Clinical Relevance:**

These findings suggest that ADPC-PRP therapy should be considered for dogs with ST.

## Introduction

Supraspinatus tendinopathy (ST) is a common cause of forelimb lameness in dogs. The etiology of ST is thought to be repeated strain activity and overuse from chronic repetitive activity, with a failure of adequate remodeling (1–4). As in humans, it can be challenging to treat and recurrence is not uncommon. While inflammation may play a role in the initiation of ST, it generally is not involved in the propagation and progression of the disease process. Histology of pathologic supraspinatus tendons shows either absent to minimal inflammation with hypocellularity, a loss of tightly bundled collagen appearance, an increased proteoglycan content, and a lack of neovascularization in response to injury (4–13). Tendons damaged from repeated strain demonstrate discontinuous, disorganized tendon fibers with little to no inflammation, occasional mineralization within the tendon, and bony remodeling at its insertion site in chronic cases (5, 12). In chronic cases, calcification at the site of insertion has been well documented in both humans and dogs (8, 13–16).

Diagnostic musculoskeletal ultrasonography is a relatively new technique that has been used and validated for the diagnosis of ST in both humans and dogs (1, 17–24). Musculoskeletal ultrasound provides a non-invasive definitive diagnosis of ST, and allows for facile and cost-effective sequential examinations to assess response to treatment. Changes in size, shape, and echogenicity of the tendon found on diagnostic ultrasound all may indicate ST (25).

Previous reports on the treatment of ST in dogs have included both conservative and surgical management (26–30). While both surgical management and conservative management have been reported for treatment of ST in dogs, up to 55% of surgically treated dogs and in up to 33% of non-surgically treated dogs have persistence or recurrence of lameness (26, 27). Regarding conservative medical management, our retrospective study on 327 ST cases in dogs revealed 75% failed to respond to rest and non-steroidal anti-inflammatory drugs and 40% failed to respond to a dedicated rehabilitation therapy program (30). Recent studies have suggested the potential efficacy of biologic regenerative therapies in humans (31, 32). One recent study in which a single platelet-rich plasma injection for ST was performed in 10 dogs showed a subjective (owner-assessed) improvement in lameness and function in 40% of dogs with improved tendon heterogenicity and in 60% of dogs with improved echogenicity (33). However, to the authors’ knowledge no studies have evaluated the use of stem cell therapy in treating ST in dogs.

Mesenchymal stem cells (MSC) found in bone marrow and adipose tissue can differentiate into multiple cell lines, including bone, cartilage, and fibrous connective tissue, such as tendons (34). In addition, MSC cells secrete cytokines and growth factors that reduce inflammation, inhibit programmed cell death in the cells within the tissue, recruit circulating stem cells to the area, and integrate and remodel the tissue through a well described paracrine effect (35). Meta-analysis of MSC effects on tendon healing suggests that stem cells increase collagen fiber density, enhance tissue architecture, restore a nearly normal tendon–bone interface, and improve biomechanical strength (36). In addition to the effects of MSCs on tendons, there is evidence demonstrating that MSCs survive when extracted and placed into the tendon environment and that cells can be stored for later use. Although the evidence for use of stem cells in tendons remains limited due to low clinical trial data, dozens of pre-clinical studies strongly support its potential role in tendon healing (37).

Mesenchymal stem cells have potent anti-inflammatory, anti-fibrosis, pro-angiogenic properties, and can integrate into tendon tissue and contribute to healing (38). MSCs require growth factor supplementation for growth *in vitro*, and the tendinopathy environment may not allow for optimal MSC cell growth and integration. Moreover, MSCs prefer to connect into a three-dimensional fibrous environment (39). PRP can provide both growth factors to promote MSC engraftment, as well as a fibrin scaffold for MSCs to attach to upon injection and platelet activation. PRP has been shown to release its growth factors 5 days after activation, while MSCs have been demonstrated to survive at the injection site at least 30 days after injection, thus giving both an early and sustained stimulus for healing (39). Synergy between stem cells and PRP has been reported (40–42). Certain growth factors and cytokines released from platelets bind to receptors on the surface of stem cells and initiate a cascade involving signal transduction, gene expression and stem cell proliferation, migration, and differentiation. In addition, PRP provides a delivery vehicle and three-dimensional scaffold to support cell survival and proper differentiation (43). For these reasons, platelet-rich plasma combination therapy together with MSCs was used in this study.

The purpose of this retrospective study was to describe the effects of adipose-derived progenitor cells and platelet-rich plasma (ADPC-PRP) combination therapy for the treatment of ST in dogs.

## Materials and Methods

### Dogs

In accordance with AAALAC International Rules of Accreditation, this study was performed with the approval of the VOSM Research Committee and with owner consent. All clients volunteered their dog for the study and provided written consent as required by Veterinary Orthopedic and Sports Medicine Group for every study participant. Medical records of client-owned dogs diagnosed with ST that were treated with ADPC-PRP combination therapy were reviewed from 2006 to 2013. Data collected included signalment, history, limb involvement, prior treatments, physical and orthopedic examination, objective temporospatial gait analysis findings, diagnostic imaging results [radiography, magnetic resonance imaging (MRI), musculoskeletal ultrasonography], arthroscopy findings, presence of concurrent shoulder and elbow pathologies, treatments administered, and outcome. Dogs were excluded from this study if there was the presence of concurrent orthopedic or neurological disease, recommended treatment and therapy plans were not adhered to, or if follow-up information was not available.

### Orthopedic Evaluation

All patients were evaluated with an orthopedic and neurological evaluation at baseline as well as follow-up at 30, 60, and 90 days post treatment. Shoulder range of motion in flexion, extension, and abduction were recorded. Discomfort was noted if found on shoulder manipulation or direct palpation of the supraspinatus. All other abnormal orthopedic or neurological findings were also recorded.

### Diagnostic Imaging

#### Radiography

Routine lateral and craniocaudal radiographs were performed of both shoulders and elbows in all patients at baseline. All radiographs were evaluated for evidence of shoulder and/or elbow pathology by a board certified veterinary surgeon.

#### Magnetic Resonance Imaging

Magnetic resonance imaging was recommended for all patients. If elected, patients were placed under general anesthesia and images of the affected shoulder were acquired using a 1.5-T MRI. All images were reviewed by a board certified veterinary radiologist.

#### Musculoskeletal Ultrasonography

**Figure 1:**
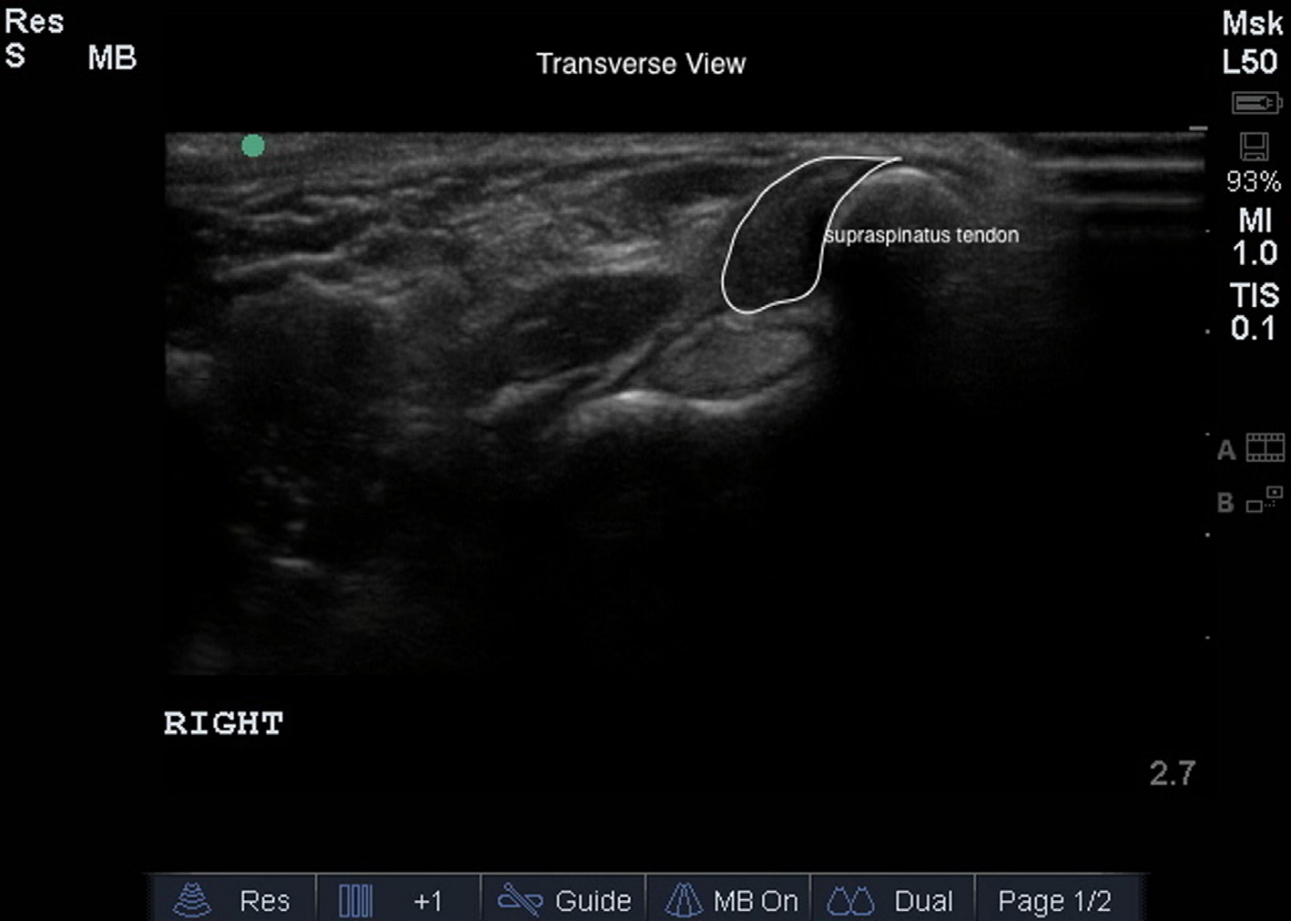
**The transverse diagnostic musculoskeletal ultrasound image of the supraspinatus tendon (white outline)**.

**Figure 2:**
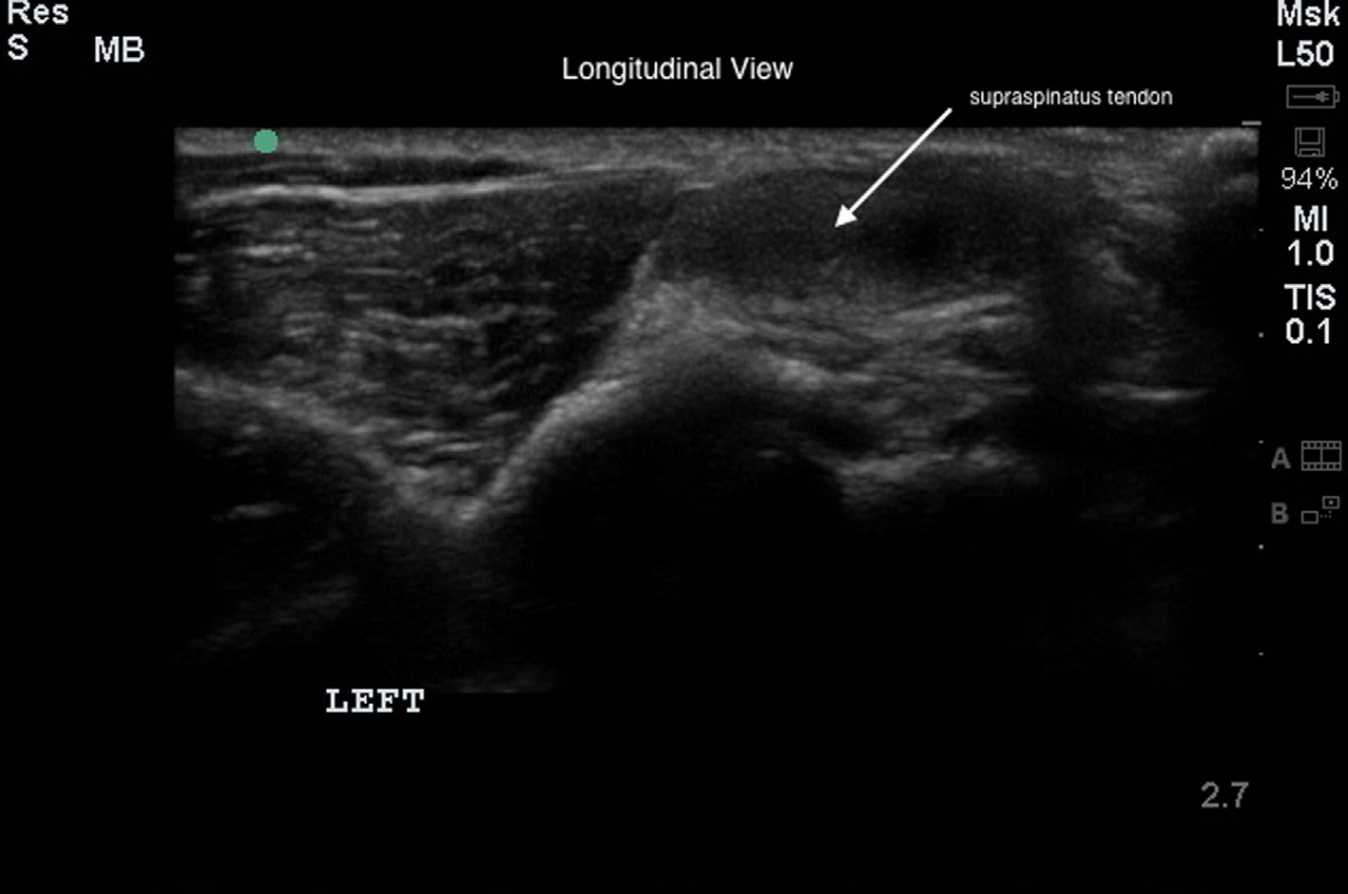
**The longitudinal diagnostic musculoskeletal ultrasound image of the supraspinatus tendon (white arrow)**.

Bilateral shoulder musculoskeletal diagnostic ultrasound was performed at baseline and at 45 and 90 days post treatment. Bilateral shoulder musculoskeletal diagnostic ultrasonographic measurements for each dog were performed using Sonosite Edge musculoskeletal ultrasound machine with a 15-6 MHz linear probe. Patients were placed in lateral recumbency, and both shoulder areas were clipped and cleaned. Sedation or anesthesia was not required. Ultrasonographic images of the supraspinatus tendon of both limbs, including transverse and longitudinal images, were obtained (Figures 1 and 2). Supraspinatus muscle depth was measured at the level of the proximal acromion to evaluate atrophy. The supraspinatus tendon cross-sectional area (CSA) was measured in the transverse plane in three images/tendon.

### Gait Analysis

Objective gait analysis was performed at baseline as well as follow-up at 90 days post treatment. Objective gait analysis was performed in a quiet room using a temporospatial pressure sensing walkway. The walkway system was equipped with a 8.23 m × 0.85 m portable mat containing 29,952 encapsulated sensors.^1^ The active dimensions of the mat were 8.04 m × 0.61 m. A 1.25 m × 0.85 m section of inactive mat was placed at each end of the walkway system to provide transition surfaces when entering and exiting the system. The mat was calibrated by the manufacturer. The walkway system interfaced with a computer and software program for processing and storage of raw data recorded from quadruped gait analysis.^2^ One camera was positioned at a height of 50 cm at the end of the walkway system to record movement.^3^ Digital video files of each pass across the walkway system were automatically linked to the data files for footfall verification.

Every dog was handled by the same examiner and allowed to acclimate to the room prior to data collection. Dogs were walked on the mat until they appeared relaxed (~4–6 passes/dog). Three passes across the walkway were recorded at both the walk and the trot. A pass was defined as a dog moving along the length of the portable walkway system in one direction and consisted of 4–6 gait cycles. Inclusion criteria for a pass were that the dog was at a walk (velocity, 0.9–1.2 m/s) or a trot (velocity, 1.8–2.6 m/s), had minimal head turning, and that gait cycle velocities did not vary by more than 10%. The first three passes that met the inclusion criteria were analyzed for each dog. Videos of each pass were reviewed to ensure that all inclusion criteria were met.

The software program was used to identify the paw print of each footfall. The software program then performed analysis of multiple variables for each pass. Data gathered or calculated from each pass provided the total pressure index percentage (TPI%) for each foot (the sum of peak pressure values recorded from each activated sensor by a paw during mat contact/total sum of peak pressure values for all feet × 100) (44). The TPI% of the injured limb was compared to the contralateral limb TPI% at baseline and at follow-up at 30, 60, and 90 days post treatment.

### Diagnostic Elbow and Shoulder Arthroscopy

Diagnostic elbow and shoulder arthroscopy was performed to rule out the presence of concurrent intra-articular pathology. A standard medial portal was utilized for arthroscopic evaluation of the elbow. Following routine aseptic preparation of the elbow, a 22-gage needle was placed into the elbow in the medial compartment. Synovial fluid was aspirated using a 5-ml syringe to ensure the needle was in the joint. Approximately 5 ml of saline was then injected into the elbow to distend the joint. A 15 blade was used to create a stab incision through the skin to allow for insertion of the cannula and trochar into the elbow. The 1.9-mm arthroscope was then inserted into the cannula. Arthroscopic evaluation was performed to confirm no signs of pathology.

A standard lateral portal was utilized for arthroscopic evaluation of the affected shoulder. Following aseptic preparation, a 22-gage needle was placed into the shoulder. Synovial fluid was aspirated using a 5-ml syringe to ensure that the needle was in the joint. Approximately 5–10 ml of saline was then injected into the shoulder to distend the joint. A 15 blade was used to create a stab incision through the skin to allow for insertion of the cannula and trochar into the shoulder. The 1.9-mm arthroscope was then inserted into the cannula. The shoulder was then assessed without the fluid pump activated in order to observe the joint in a more natural environment without significant distension. This would allow for identification of a supraspinatus bulge. The biceps, subscapularis tendon, MGL, joint capsule, and cartilage were observed to confirm no evidence of pathology.

### Falciform Collection for ADPC Processing

Patients were pre-medicated with 0.1 mg/kg hydromorphone intramuscularly, 0.2 mg/kg midazolam intramuscularly, and 0.5 mg/kg famotidine subcutaneously. General anesthesia was induced using intravenous propofol to affect. The abdomen was clipped and aseptically prepared for surgery. A 4-cm incision was made in midline in the cranial abdomen. A 2-cm incision was made through the linea. The falciform ligament was identified. Approximately 20 g of falciform adipose tissue was collected and placed into a sterile container with Delbecco’s modified Eagle’s medium (DMEM) supplemented with fetal bovine serum (FBS), penicillin, and streptomycin. The sample was then prepared for shipment. A routine closure was performed.

### Blood Collection for PRP Processing

The jugular vein was clipped and aseptically prepared. Approximately 30 ml of blood was collected using an 18-gage butterfly needle into a syringe with 5 ml of CPDA anti-coagulant. The sample was then prepared for shipment.

### ADPC and PRP Processing

Adipose and blood samples were shipped in validated containers at 4°C to Virginia Tech’s Regenerative Medicine Research Laboratory for processing as per US patent application number #62050792. Adipose was processed using established techniques (45). The tissue was mechanically and enzymatically separated to release ADPCs. The cells were then washed in phosphate-buffered saline (PBS) and nucleated cells were counted and cultured in stem cell media at 37°C in a 5% CO_2_ incubator with 95% humidity. Adherent cells were monitored daily for adherence, growth, and phenotype. Once 70% confluent, these first passage cells were detached from the flasks, washed in PBS, and suspended in autologous PRP.

Platelet-rich plasma was prepared from anti-coagulated blood via centrifugation first for 10 min at 800 g, then the platelet-rich supernatant above the buffy coat was centrifuged at 4,000 × *g* for 10 min and the pellet was resuspended in plasma to obtain a three- to fourfold increase in platelets and 80–90% reduction in white blood cells over whole blood. Platelet and white blood cell counts were performed for each sample to confirm appropriate concentrations prior to use, and prepared in 1-ml dose/tendon with 5 million ADPCs/milliliter. ADPC-PRP was then shipped back to VOSM for injection at 4°C using validated shipping containers.

### Ultrasound Guidance of ADPC-PRP Therapy

**Figure 3:**
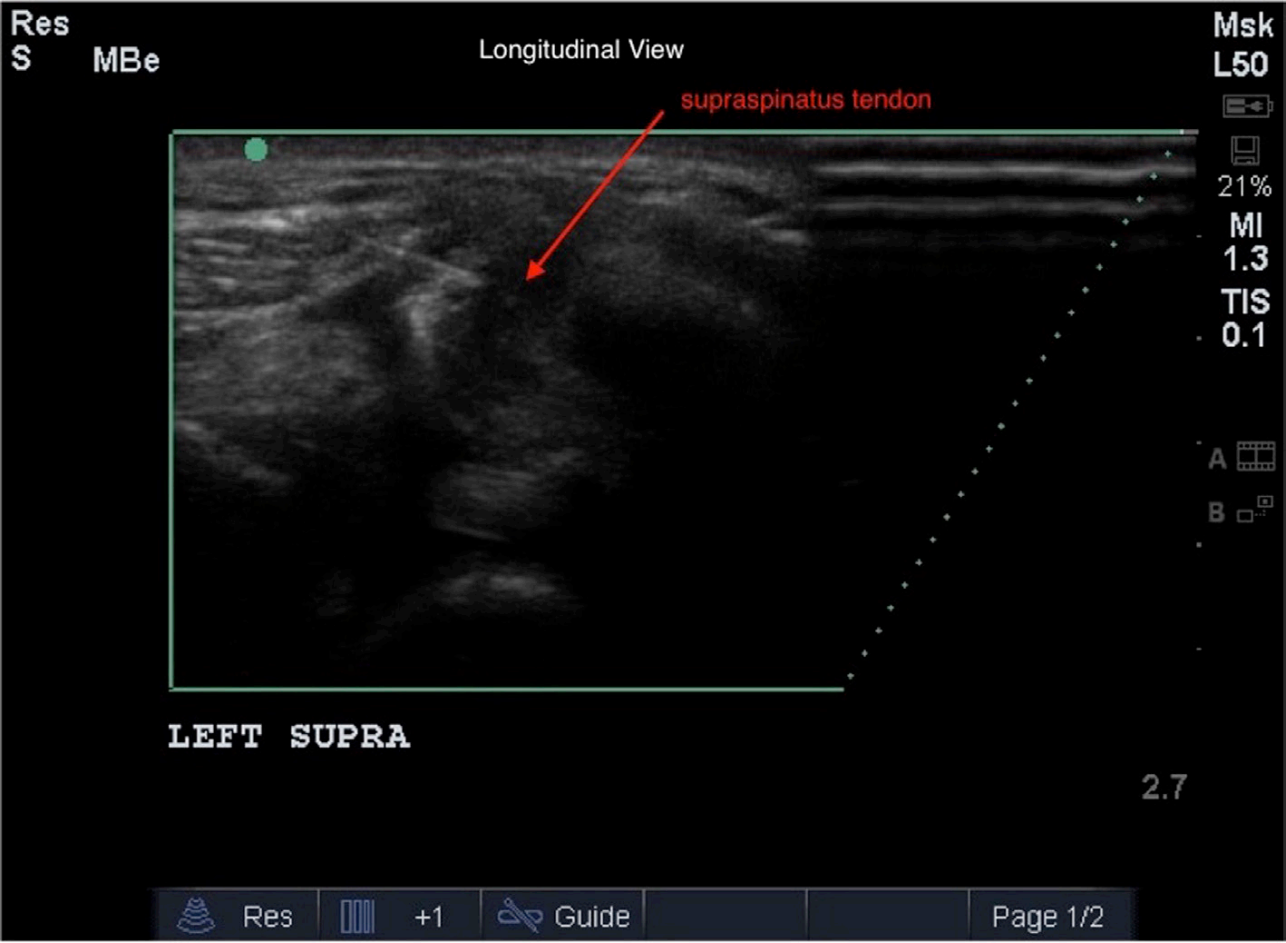
**Ultrasound guidance was used to administer ADPC-PRP therapy with a fenestration technique**. A 22-gage needle was inserted along the long axis of the supraspinatus tendon (red arrow), parallel to the ultrasound transducer. Once the needle was identified in the plane, the needle was advanced into the tendon lesion.

Ultrasound guidance was used to administer ADPC-PRP therapy with a fenestration technique (46, 47). Patients were sedated using 0.05 mg/kg of dexdomitor and 0.2 mg/kg of butorphanol intravenously. The shoulder was clipped and aseptically prepared. A 22-gage, 1.5″ needle was inserted along the long axis of the tendon, parallel to the ultrasound transducer. Once the needle was identified in the plane, the needle was advanced into the tendon lesion. As the needle was slowly passed into the lesion and withdrawn, ADPC-PRP was injected into the lesion (Figure 3). Proper placement and delivery of the ADPC-PRP was monitored continuously using ultrasound guidance.

### Post Treatment Rehabilitation Therapy

All patients were enrolled in rehabilitation therapy following ADPC-PRP therapy. For the first 8 weeks, each patient participated in once weekly manual/massage therapy, class IIIb low-level laser therapy for the affected shoulder (5 J/cm^2^), and a twice daily at home exercise program. Additionally, at 8 weeks following treatment, patients were allowed to start hydrotherapy with underwater treadmill. No NSAIDs, corticosteroids, class IV low-level laser therapy, therapeutic ultrasound, or TENS/NMES were permitted at any time during the first 8 weeks, nor were any of these treatments required during the remaining recovery period in any of the patients.

### Statistics

Statistical analysis of CSA over time and TPI over time was performed using repeated measures ANOVA with *post hoc* Tukey’s test. Significance was set at *P* < 0.05.^4^

## Results

### Patient Demographics

Medical records of 55 dogs diagnosed with unilateral ST treated with ADPC-PRP therapy were reviewed. Age ranged from 1 to 14 years (average 6.4 years, median 6 years). No sex predisposition was apparent with 26 female dogs (1 intact) and 29 male dogs (2 intact). There were 13 Border Collies, 13 Labrador Retrievers, 7 German Shepherd Dogs, 5 Mixed Breed Dogs, 3 Boxer Dogs, 3 Welsh Corgis, 3 Golden Retrievers, 2 Bernese Mountain Dogs, and 1 of each of the following: Basenji, Greater Swiss Mountain Dog, Standard Poodle, Rhodesian Ridgeback, Rottweiler, and Wheaton Terrier (Table 1). Occupation was evenly split between companion dog and performance/sporting dog at 50.1 and 49.1%, respectively (Table 2). Duration of lameness ranged from 1 week to over 1 year. A majority of dogs, 61.8%, had previously failed to respond to non-steroidal anti-inflammatory drugs and 45.5% also failed rehabilitation therapy. Of the performance/sporting dogs, 96.4% of them returned to sport after 4 months following ADPC-PRP therapy.

**Table 1:** **Patient breed**.

Breed	Number of patients
Basenji	1
Bernese Mountain Dog	2
Border Collie	13
Boxer	3
Corgi	3
German Shepherd Dog	7
Golden Retriever	3
Greater Swiss Mountain Dog	1
Labrador Retriever	13
Mixed Breed	5
Poodle – Standard	1
Rhodesian Ridgeback	1
Rottweiler	1
Wheaton Terrier	1

**Table 2:** **Patient occupation**.

Occupation	% of patients
Agility	37
Field trial	7.4
Flyball	7.4
Herding	7.4
Hunting	3.7
Obedience	18.5
Rally	7.4
Show	11.1

### Radiographs

**Figure 4:**
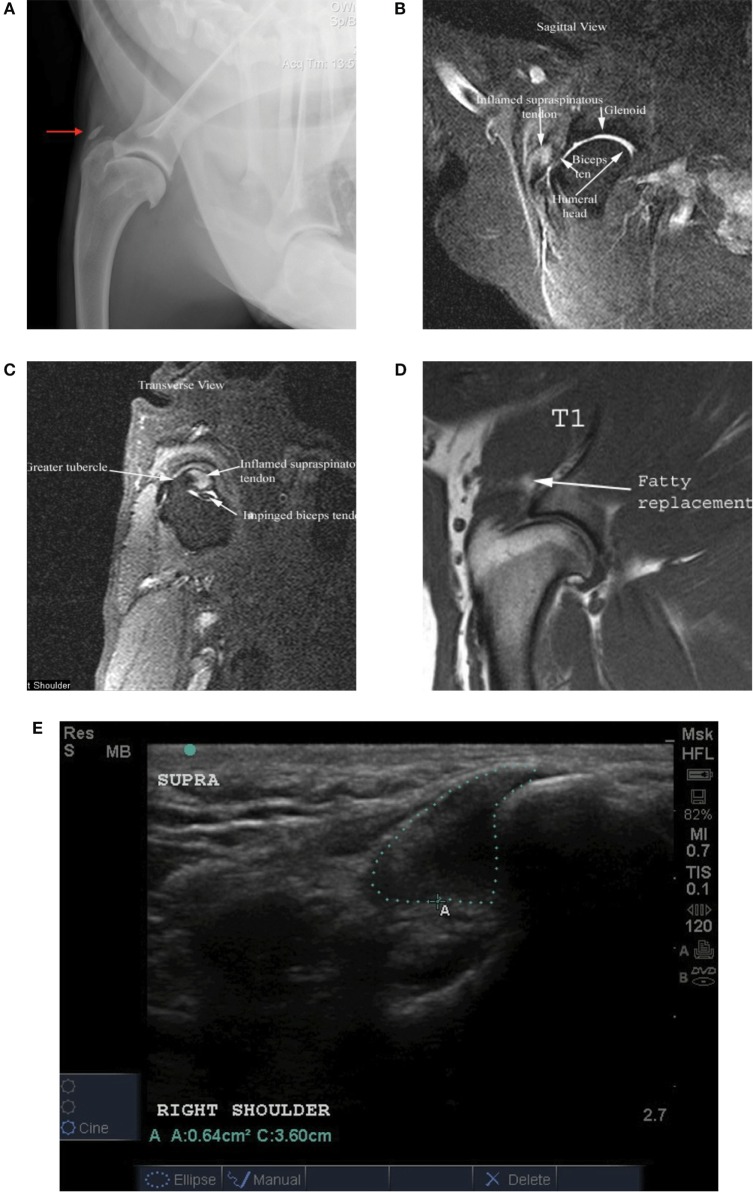
**(A)** Shoulder radiograph with mineralization of the supraspinatus tendon insertion (red arrow); **(B)** STIR sagittal image of a shoulder MRI in a patient with a supraspinatus tendinopathy showing an inflamed supraspinatus tendon; **(C)** STIR transverse image of a shoulder MRI in a patient with a supraspinatus tendinopathy showing a flattened or oval appearance of the biceps tendon and loss of fluid around the biceps tendon within the bicipital groove at the level of insertion of the supraspinatus; **(D)** T1 MRI image in a patient with a supraspinatus tendinopathy showing fatty replacement at the myotendinous junction of the supraspinatus tendon; **(E)** diagnostic musculoskeletal shoulder ultrasound image depicting an enlargement of the supraspinatus tendon.

Routine lateral and craniocaudal radiographs were performed of both shoulders and elbows in all patients at baseline. Mineralization of the affected supraspinatus tendon was noted in eight cases (14.5%) (Figure 4A). No other abnormalities were noted.

### Magnetic Resonance Imaging

Magnetic resonance imaging of the shoulder was performed in 31 of the 55 cases. Of these 31 cases, findings indicative of a ST include hyperintensity of signal on T1 and STIR sequences of the ST at its insertion on the greater tubercle (Figure 4B). Mineralization can also be seen on MRI. A flattened or oval appearance of the biceps tendon, loss of fluid around the biceps tendon within the bicipital groove at the level of insertion of the supraspinatus, subsequent compartmentalization of fluid distal to supraspinatus insertion, fatty replacement at the myotendinous junction (seen on T1) of the supraspinatus and/or displacement of the biceps tendon from the bicipital groove all characterized biceps impingement secondary to ST (Figures 4C,D).

### Musculoskeletal Diagnostic Ultrasound

Cross-sectional area of the treated (ADPC-PRP injected) tendon was compared to the contralateral (non-affected) tendon at baseline and at 90 days post treatment.

**Figure 5:**
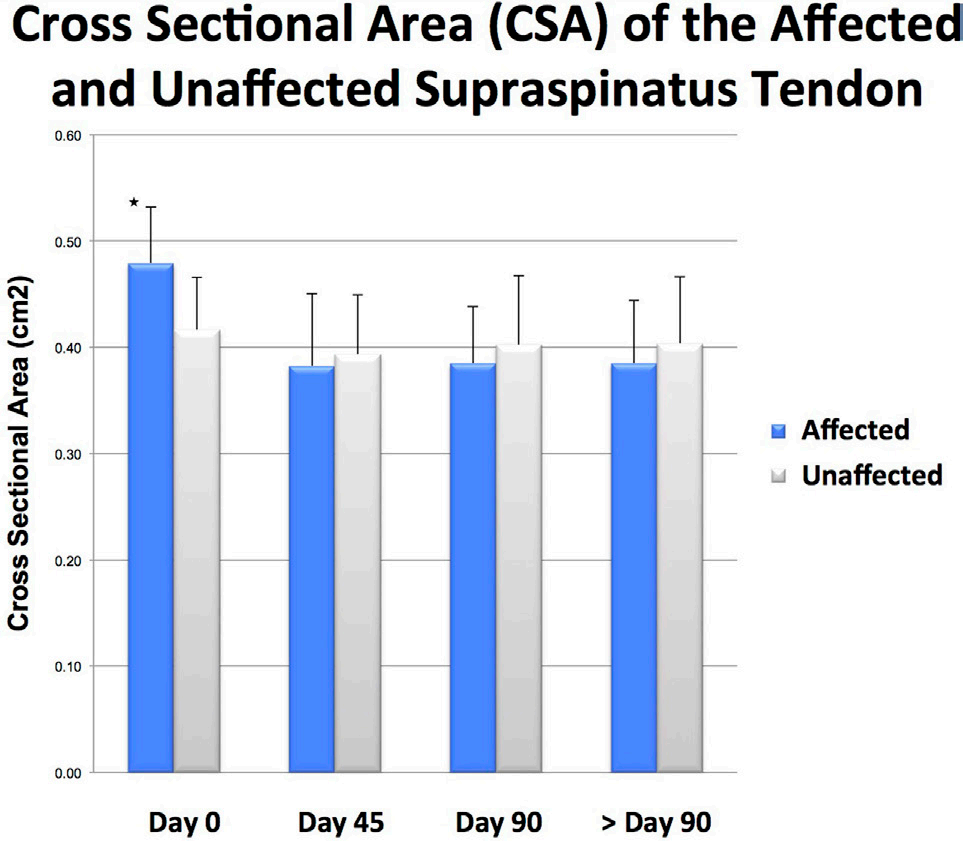
**The total pressure index percentage (TPI%) of the affected limb in individual patients at days 0 and 90**.

**Figure 6:**
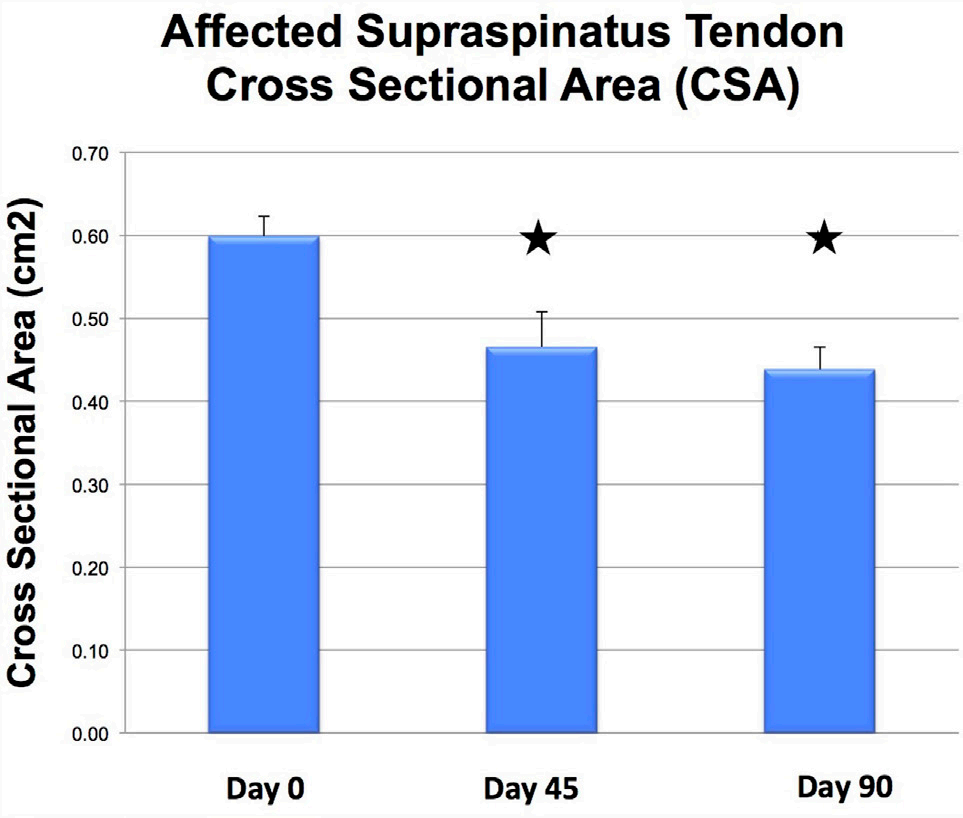
**Results of objective gait analysis showed a significant difference in total pressure index percentage (TPI%) between the injured and contralateral normal forelimb at day 0 (*p* = 0.014)**. There was no significant difference in total pressure index percentage (TPI%) between the injured and contralateral normal forelimb at day 90.

**Figure 7:**
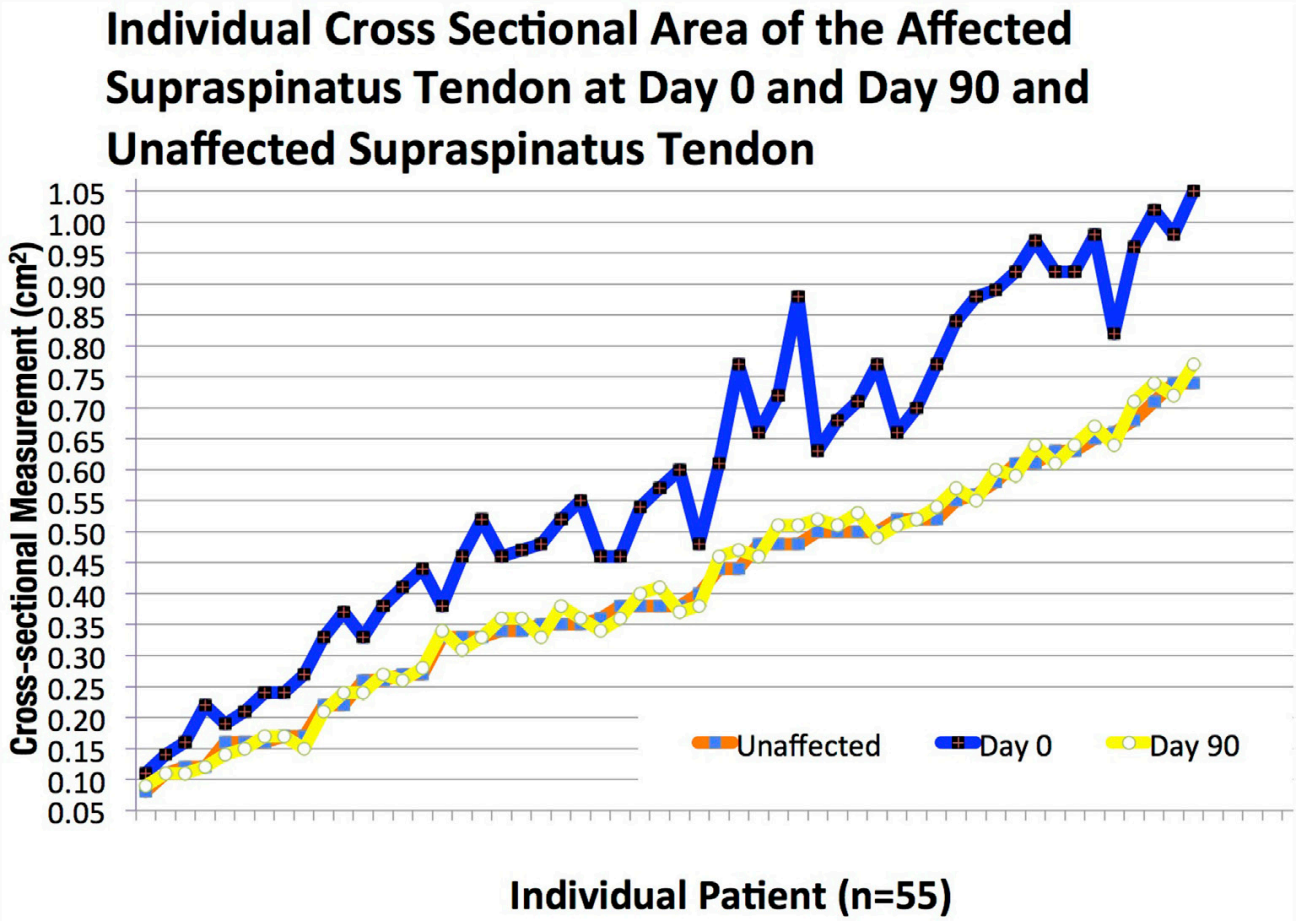
**There was a significant difference in cross-sectional area (CSA) noted between the injured and contralateral normal supraspinatus tendon prior to ADPC-PRP therapy at day 0 (*p* = 0.002)**.

There was a significant difference in CSA noted between the injured and contralateral normal supraspinatus tendon prior to ADPC-PRP therapy (*p* = 0.002; Figures 4E and 5). No significant difference in CSA was noted between the injured and contralateral normal supraspinatus tendon at 45 and 90 days post treatment (Figure 5). Following treatment, a significant reduction in tendon size (CSA) was noted in the treated tendon at both 45 and 90 days following treatment when compared to the initial CSA (*p* = 0.001; *p* = 0.005; Figures 6 and 7). Reduction in supraspinatus size was noted in all cases with 82% of cases reaching the contralateral supraspinatus size. All cases showed improvement in fiber pattern of the affected supraspinatus tendon at both 45 and 90 days following treatment.

### Objective Gait Analysis

**Figure 8:**
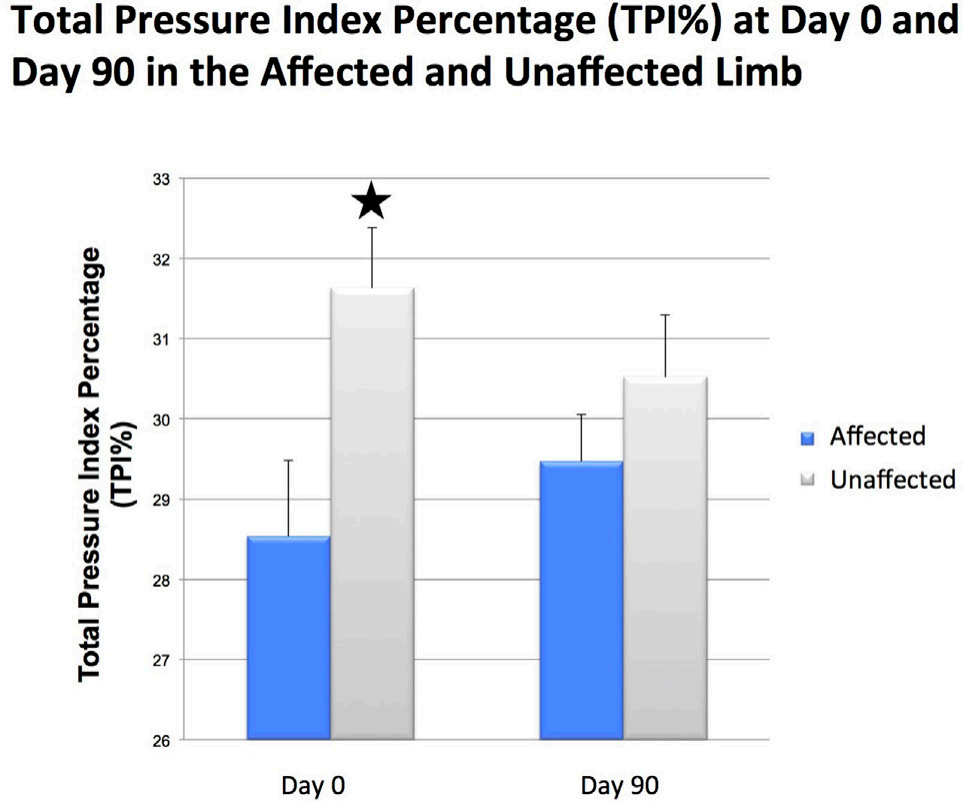
**Following treatment, a significant reduction in tendon size (CSA) was noted in the treated tendon at both 45 and 90 days following treatment when compared to the initial CSA (*p* = 0.001; *p* = 0.005)**.

**Figure 9:**
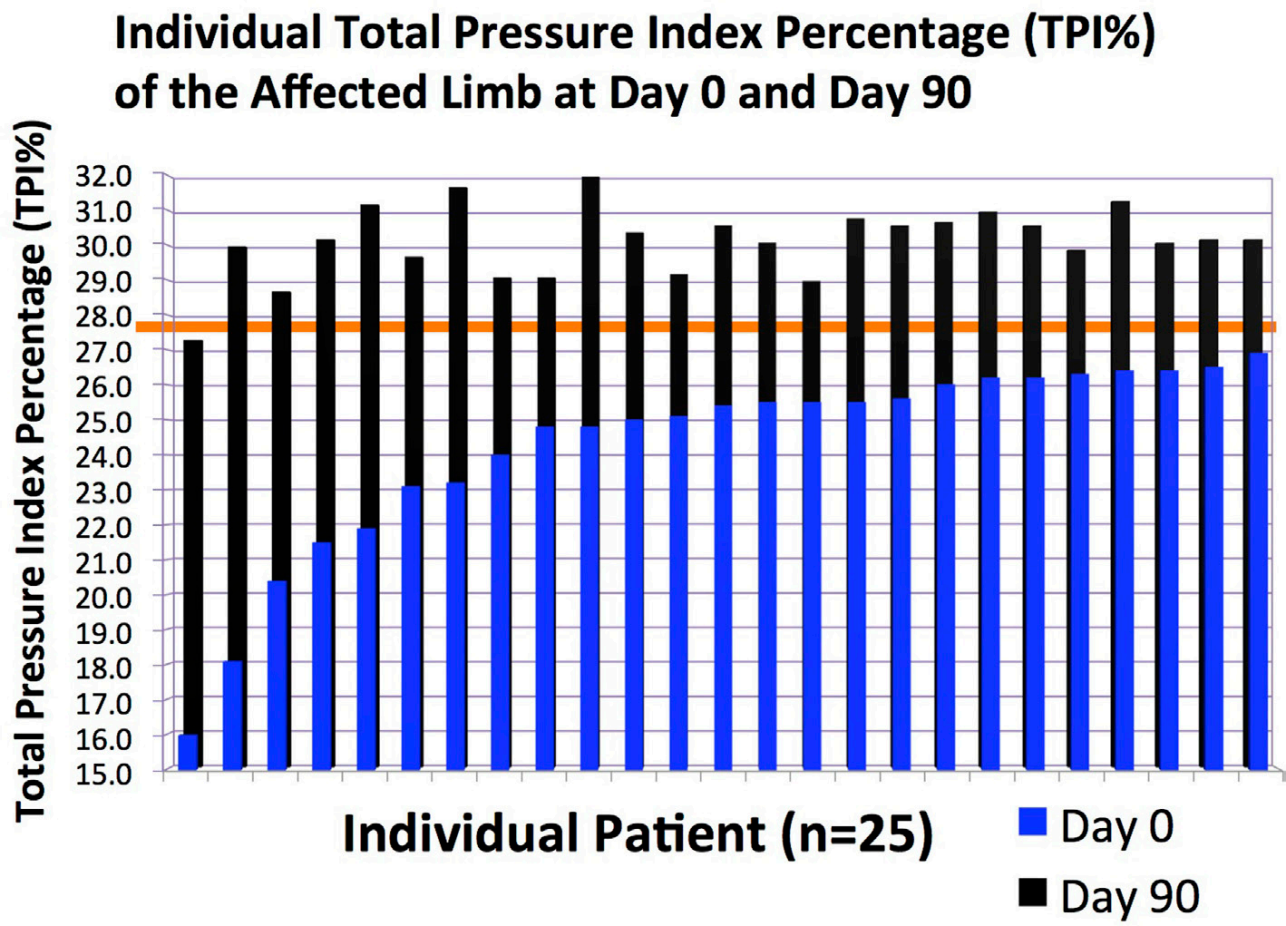
**The individual cross-sectional area (CSA) of the affected supraspinatus tendon at days 0 and 90 and unaffected supraspinatus tendon**.

Following ultrasound-guided injection of ADPC-PRP therapy, objective gait analysis was available on 25 of the 55 dogs at 90 days post ADPC-PRP therapy. TPI% of the treated forelimb was compared to the contralateral (non-affected) forelimb at baseline and at each follow-up evaluation. Results of objective gait analysis showed a significant difference in TPI% between the injured and contralateral normal forelimb prior to injection with the injured limb having a lower TPI% (*p* = 0.014; Figure 8). Following treatment, a significant increase in TPI% was noted in the injured (treated) forelimb at 90 days post treatment (*p* = 0.036). At 90 days following treatment, 88% of cases had no significant difference in TPI% of the injured limb to the contralateral limb (Figures 8 and 9).

## Discussion

Based on the objective gait analysis and diagnostic ultrasound results, ADPC-PRP therapy appears to be promising for dogs with ST, especially those who have failed to respond to conservative management and rehabilitation therapy. Measurable improvement in lameness as indicated by TPI, and in CSA, was seen over time and/or in comparison to contralateral normal tendons.

When considering treatment options for ST patients, it is important to note that many of the patients failed to respond to conservative management prior to presentation as 61.8% of patients failed to respond to NSAID therapy and 45.5% failed to respond to rehabilitation therapy. This may suggest that conservative management is often insufficient to treat ST. While dogs treated surgically have been shown to improve in gait and overall comfort, regenerative medicine therapy also shows promising results and should be considered for dogs with ST. Future studies are needed to further evaluate the use of regenerative medicine therapy for ST and other tendon injuries in the canine.

Limitations of this study are primarily related to the nature of a retrospective study, and the use of a combination therapy. Future studies should be randomized, blinded, and controlled. While there was no placebo control, 61.8% had previously failed to respond to NSAID therapy and 45.5% had previously failed rehabilitation therapy. Initially, our group treated refractory cases of ST with PRP alone. This treatment in a limited number of cases subjectively did not seem to improve healing by ultrasonographic evaluation, and PRP therapy was abandoned in favor of the described ADPC-PRP therapy. In contrast to PRP alone, initial cases treated with ADPC/PRP therapy showed subjective improvement in both echogenicity and lameness. A small pilot study using a leukocyte and platelet-rich plasma alone to treat ST in 10 dogs improved lameness and function (33). It is possible that other formulations of PRP with higher white blood cell and/or platelet concentrations may be more efficacious than the leukocyte reduced or pure PRP used in our cases (48). It is also possible that PRP treatment has an effect on pain, but does not promote remodeling resulting in similar ultrasonographic appearance, but improvement in lameness as seen in Ho et al.’s study (33). Finally, this study did not include immunohistochemistry, immunofluorescence, and histopathology to confirm that the stem cells have differentiated and incorporated into the injured tissues. Further studies would be needed to definitively prove the differentiation and engraftment of the stem cells.

In conclusion, ADCP in combination with PRP may provide an adequate biologic effect leading to tendon healing and improved function. Because of the limited efficacy of current non-surgical management, consideration should be given to the use of regenerative medicine interventions in tendons that demonstrate poor natural healing potential.

## Author Contributions

We certify that all authors meet the qualifications for authorship as listed below: (1) substantial contributions to the conception or design of the work or the acquisition, analysis, or interpretation of data for the work; (2) drafting the work or revising it critically for important intellectual content; (3) final approval of the version to be published; (4) agreement to be accountable for all aspects of the work in ensuring that questions related to the accuracy or integrity of any part of the work are appropriately investigated and resolved.

## Conflict of Interest Statement

The authors declare that the research was conducted in the absence of any commercial or financial relationships that could be construed as a potential conflict of interest.

## Footnotes

^1^GaitFour Pressure Sensing Walkway, platinum version, CIR Systems Inc., Harvertown, PA, USA.

^2^GaitFour, platinum version, CIR Systems Inc., Harvertown, PA, USA.

^3^Logitech MP web camera, Logitech, Freemont, CA, USA.

^4^GraphPad Prism 6, GraphPad Software Inc., La Jolla, CA, USA.

## Abbreviations

ADPC, adipose-derived progenitor cells; MSC, mesenchymal stem cells; PRP, platelet-rich plasma; ST, supraspinatus tendinopathy; TPI%, total pressure index percentage.
